# Treatment of COVID-19 Pneumonia: the Case for Placenta-derived Cell Therapy

**DOI:** 10.1007/s12015-020-10004-x

**Published:** 2020-07-21

**Authors:** Ekaterine Berishvili, Laurent Kaiser, Marie Cohen, Thierry Berney, Hanne Scholz, Yngvar Floisand, Jonas Mattsson

**Affiliations:** 1 Cell Isolation and Transplantation Center, University of Geneva School of Medicine, Geneva, Switzerland; 2 Institute of Medical and Public Health Research, Ilia State University, Tbilisi, Georgia; 3 Cell Isolation and Transplantation Center, Centre Médical Universitaire, 1, rue Michel-Servet, CH-1211 Geneva 4, Switzerland; 4 Division of Infectious Diseases, Virology Laboratory and Geneva Centre for Emerging Viral Diseases, University of Geneva Hospitals, Geneva, Switzerland; 5 Department of Pediatrics, Gynecology and Obstetrics, University of Geneva School of Medicine, Geneva, Switzerland; 6 Division of Transplantation, University of Geneva Hospitals, Geneva, Switzerland; 7 Department of Transplant Medicine, Department of Cellular Therapy, University of Oslo, Oslo, Norway; 8 Centre of Excellence, Institute of Basic Medical Sciences, University of Oslo, Oslo, Norway; 9 Department of Hematology, Oslo University Hospital, Oslo, Norway; 10 Center for Cancer Cell Reprogramming, Institute for Cancer Research, Oslo University Hospital, Oslo, Norway; 11 Gloria and Seymour Epstein Chair in Cell Therapy and Transplantation, University of Toronto, Toronto, Ontario Canada

## Abstract

Nearly 500’000 fatalities due to COVID-19 have been reported globally and the death toll is still rising. Most deaths are due to acute respiratory distress syndrome (ARDS), as a result of an excessive immune response and a cytokine storm elicited by severe SARS-CoV-2 lung infection, rather than by a direct cytopathic effect of the virus. In the most severe forms of the disease therapies should aim primarily at dampening the uncontrolled inflammatory/immune response responsible for most fatalities. Pharmacological agents - antiviral and anti-inflammatory molecules - have not been able so far to achieve compelling results for the control of severe COVID-19 pneumonia. Cells derived from the placenta and/or fetal membranes, in particular amniotic epithelial cells (AEC) and decidual stromal cells (DSC), have established, well-characterized, potent anti-inflammatory and immune-modulatory properties that make them attractive candidates for a cell-based therapy of COVID19 pneumonia. Placenta-derived cells are easy to procure from a perennial source and pose minimal ethical issues for their utilization. In view of the existing clinical evidence for the innocuousness and efficiency of systemic administration of DSCs or AECs in similar conditions, we advocate for the initiation of clinical trials using this strategy in the treatment of severe COVID-19 disease.

## Introduction

Since December 2019, when it was first tracked in China, the novel human coronavirus SARS-CoV-2, the agent of COVID-19 disease, has quickly spread to pandemic proportions, with rapid person-to-person transmission, and has become a global health emergency. The virus has shown an extremely pathogenic potential, mainly targeting frail individuals, and leading to fatal pneumonia and Acute Respiratory Distress Syndrome (ARDS). As of the time of this writing, close to 500’000 fatalities due to COVID-19 have been reported worldwide and the death toll is still rising [1].

In this opinion paper, we briefly present the pathogenesis of severe COVID-19 disease, argue that it should be treated primarily with an anti-inflammatory strategy, and propose that this can be achieved by cell therapy using placenta-derived cells known for their anti-inflammatory and immunomodulatory properties, as previously successfully attempted in similar diseases.

## COVID-19: a 2-step Clinical Course

**Fig. 1:**
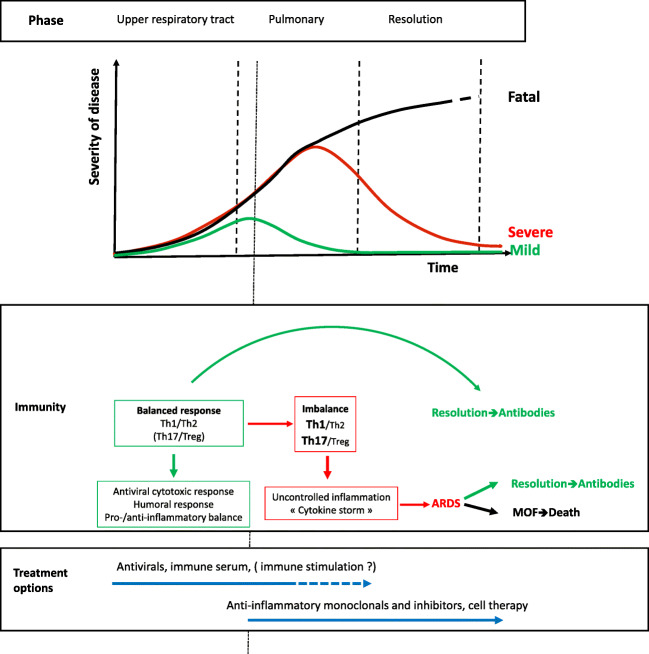
Schematic representation of COVID-19 clinical course and treatment options. Viral infection initiates in the upper respiratory tract, where it causes mild disease. At this stage, the immune response is balanced, so as to allow cytotoxic clearance of virus-infected cells, elicit humoral response and maintain a controlled inflammatory/anti-inflammatory (Th1/Th2) balance. It may then progress to broncho-alveolar infection, where the immune response may remain balanced, and the clinical course remain mild and evolve toward resolution. In the lungs, the immune response, possibly in situations of higher viral load, may also progress to a severe uncontrolled inflammatory condition, with Th1/Th2 and Th17/Treg imbalance, recruitment of macrophages and neutrophils, and a « cytokine storm » causing ARDS and a systemic and potentially lethal disease. The severity and lethality of the disease is the consequence of this overwhelming inflammatory reaction in which antiviral drugs will not suffice to control the clinical course. There is a turning point (symbolized by a thin dotted line) from which an anti-inflammatory/immunomodulatory strategy is required to help dampen the disease. Anti-cytokine small molecules are currently being tested. We propose that cell therapy with placenta-derived immunomodulatory cells (DSCs, AECs) could be an efficient strategy at this stage of the disease

The clinical course of severe COVID-19 disease is schematically thought to follow a 2-step pattern (Fig. 1). In the first phase, viral infection usually starts in the upper respiratory tract, where it causes flu-like symptoms and elicits an adaptive immune response aiming at controlling the infection and clearing the virus. This is a stage in which antiviral drugs may be efficient at controlling the disease, but, unfortunately, we lack SARS-CoV-2-specific antivirals. The lopinavir/ritonavir combination has not shown obvious efficiency [2], encouraging results have been reported with remdesivir [3], and clinical trials testing other antiviral candidate therapies are ongoing, including antibody therapy using sera from convalescent COVID-19 patients [4].

If the immune system is unable to mount a response strong enough to clear the virus at this early stage, infection can progress from upper respiratory tract infection to pneumonia, in which alveolar epithelial cells get infected. In the lungs, an excessive reaction from the immune system may then overwhelm the ongoing cellular and humoral adaptive responses.

Our current understanding is that the failure to control viral replication immediately leads to an uncontrolled inflammatory reaction, and that a direct cytopathic effect of the virus is not the main driver of the severe pulmonary complications of COVID-19. There is growing evidence that a Th1- and Th17-driven reaction elicits a cytokine storm, reminiscent of secondary haemophagocytic lymphohistocytosis (sHLH), involving extravasation of blood neutrophils and excessive monocyte/macrophage activation. The uncontrolled release of proinflammatory cytokines and chemokines (IFN-γ, interleukin (IL)-1β, IL-6, TNF-α, IL-2, IL-7, IL-8, G-CSF, IP-10, MCP-1) in the lung triggers edema, dysfunction of gas exchange, ARDS, acute cardiac injury, secondary bacterial infection and, ultimately, death [5–11]. One emerging hypothesis involves activation of the NLRP3 inflammasome in SARS-CoV-2 infection as a key mediator triggering the cytokine storm. Several mechanisms have been proposed and are under investigation, and interestingly, NLRP3 inflammasome activation by the SARS-CoV virus has been demonstrated in the past [12, 13]. The systemic cytokine release syndrome may in turn hit peripheral organs, causing their irreversible damage and multi-organ failure. Atrophy of the spleen and lymph nodes with reduced lymphocyte numbers indicate failure to control the infection by a severely impaired immune system. Most of the infiltrating cells found in the lungs are monocytes and macrophages, lymphocytic infiltration being minimal [11]. ARDS is the leading cause of COVID-19-related mortality, affecting 45–62% of patients critically ill with COVID-19 pneumonia, with a median time from admission in the intensive care unit (ICU) to death ranging from 7 to 12 days [5].

## Targeting the Inflammatory Reaction in Severe COVID-19

At this stage of the COVID-19 disease, it is apparent that, once severe disease is established, purely antiviral therapy is unlikely to be sufficient and that anti-inflammatory/immunomodulatory strategies should be applied. Timely targeting of the inflammatory phenomena that are the hallmark of the disease, in an attempt to prevent the ensuing cytokine storm, appears as a valid strategy to decrease mortality, or simply time in ICU, with the secondary benefit of increasing the availability of ICU resources, beds, personnel and respirators, and thus further decreasing mortality.

Corticosteroids have been used to that purpose from the onset of the epidemic in Wuhan, but their impact is controversial and their utilization is not recommended [10]. A retrospective review of IL-6 blockade with tocilizumab has suggested that it could reduce COVID-19 mortality, but appropriate studies are still lacking [14]. A number of randomized controlled trials (RCT) are currently registered to test tocilizumab, but also other drugs targeting IL-6 (siltuximab), IL-1 (anakinra) or IFN-γ (emapalumab), janus kinase inhibitors (ruxolitinib, baricitinib) or other agents [15]. Reliable results of completed RCTs using such types of anti-inflammatory small molecules are still lacking and eagerly expected. Pharmaceutical inhibition of the NLRP3 inflammasome has more recently been proposed as a potential addition to the anti-inflammatory armamentarium to fight severe COVID-19 disease [12, 13].

In parallel with the search for a potent pharmacologic agent, alternative anti-inflammatory or immunomodulatory approaches that do not impair antiviral response and preserve from tissue damage are worth being considered. Among them, stem cell-based therapies might be an interesting option for the effective management of the cytokine storm/sHLH and modulation of the monocyte/macrophage response. Mesenchymal stromal cells (MSC) have been used for decades from basic research to clinical trials, more for their anti-inflammatory and immunomodulatory properties, than for stemness characteristics [16–18]. The efficiency of MSCs in treating ARDS induced by viral infections has been shown in a number of pre-clinical models, and their safety established in a few phase I-II clinical trials [19, 20]. Together with its lung repair and regenerative properties, MSC-based therapy might be effective in preventing or mitigating the cytokine storm, with the potential of reducing COVID-19 morbidity and mortality [21]. To this end, there are currently more than 30 registered clinical trials planning to use stem cell-based therapy for COVID-19 pneumonia and ARDS [15]: umbilical cord blood, Wharton’s jelly, menstrual blood, dental pulp, and bone marrow and adipose tissue derived stromal cells are all in the pipeline for clinical evaluation.

In a recent pilot study, in which seven COVID-19 patients with ARDS were treated with adult bone marrow-derived MSCs, the authors report improvement in both clinical and inflammatory outcome compared to a control group of 3 patients [22]. These projects and observations point to the need for trustworthy results obtained in RCTs. A clear mechanistic rationale for the selection of the cell source is also of primary importance, rather than the reliance on relatively easy manufacturing approaches and high cell yields.

Along this line, perinatal tissues may be worthy of consideration. Cells derived from the human placenta or amniotic membrane have shown therapeutic potential in terms of immunomodulatory properties and their procurement has the immense advantage of being easy, non-invasive, perennial and devoid of ethical issues.

## The Rationale for Human Placenta- and Amnion-derived Cell Therapy

The placenta and the fetal membranes serve as immunological barriers at the fetal-maternal interface in pregnancy. During pregnancy, a switch from the Th1 cytokine profile to the Th2 profile occurs to protect the fetus from the mother’s immunity. The placenta contributes to this natural evolution of maternal immunity [23]. Maternal and fetal immune cells interact in the decidua, a membrane of maternal origin that plays an important role in feto-maternal tolerance [24]. Cells isolated from different parts of placental tissues, including amnion [25, 26], decidua [26, 27], and umbilical cord have been studied for their multipotent differentiation capacities [28], but most importantly for their immunomodulatory and anti-inflammatory properties. Intact amniotic membranes from term placentas have been used for almost a century to treat severe burn injuries, and, later, corneal wounds, and are known for their ability for tissue repair without being rejected [29]. In a recent publication, a marked reduction of pulmonary fibrosis was observed by the intratracheal injection of human amniotic membrane-derived MSCs in a bleomycin-induced murine model, mediated by a modulation of lymphocyte and macrophage phenotypes [30].

**Fig. 2:**
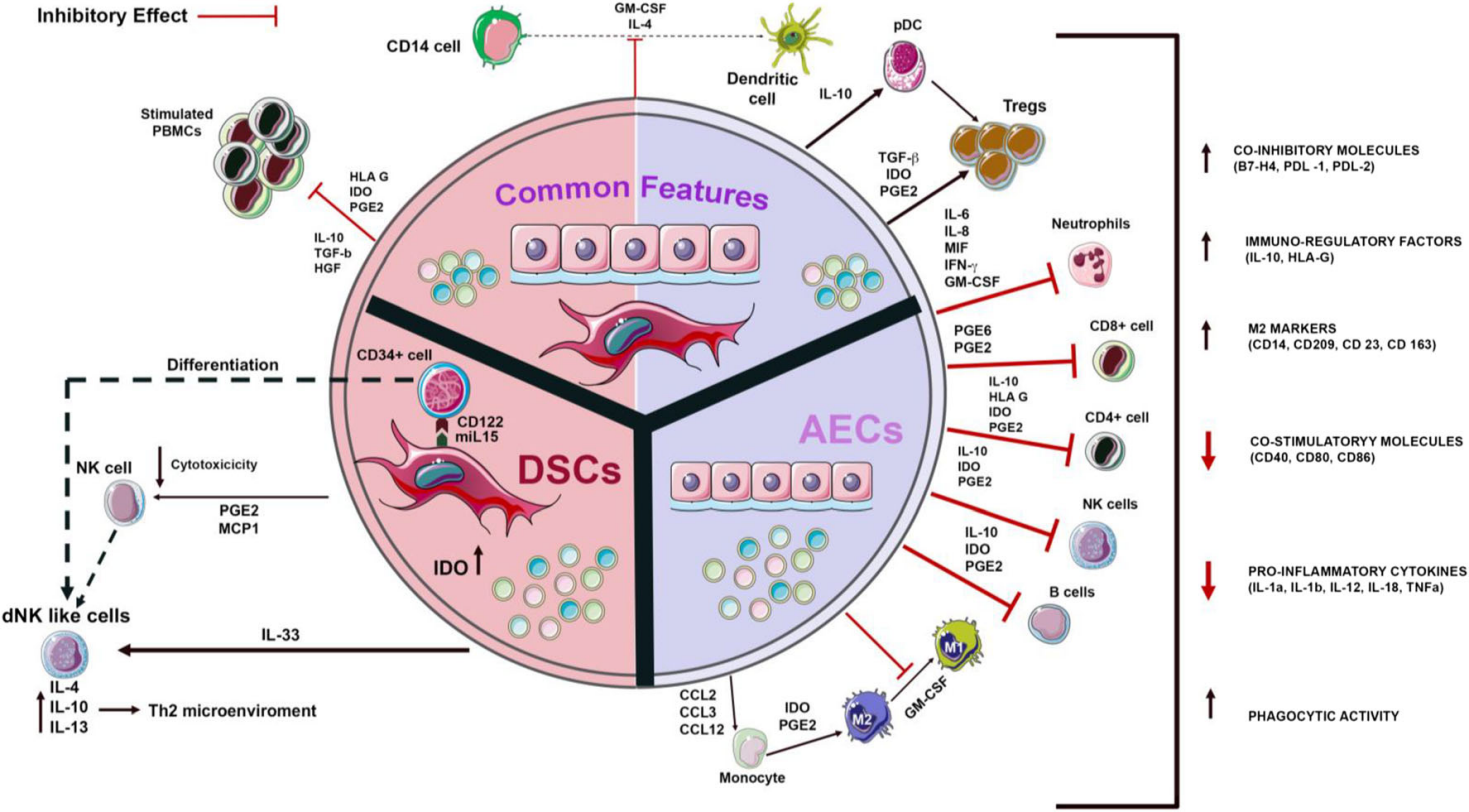
Immunomodulatory properties of DSCs and AECs. **DSCs** inhibit NK cell proliferation and promote their differentiation into a dNK phenotype via two mechanisms: (a) by the release of TGFβ, IL-10, and MCP-1 and (b) by the interaction between mIL-15 and CD122 receptor. DSCs also secrete IL-33, contributing to the establishment of a Th2 microenvironment. Finally, they inhibit monocyte differentiation into dendritic cells mediated by PGE2. **AECs** suppress proliferation, inflammatory cytokine production, and differentiation of T cells. Soluble factors secreted by AECs, including PGE2, TGF-β, Fas-L, MIF, TRAIL, and HLA-G, block dendritic cell and M1 macrophage differentiation and promote differentiation of monocytes into an anti-inflammatory M2 phenotype. AECs also modulate the host immune system, mainly through downregulation of TNF-α, IFN-γ, MCP-1 and IL-6 and upregulation of anti-inflammatory cytokines. **DSCs and AECs** also share common features: both cell types stimulate the generation of Treg cells trough PGE2, TGF-β and IDO and suppress proliferation of activated PBMCs. They also block monocyte to dendritic cell maturation AEC: amniotic epithelial cell; dNK cell: decidual natural killer cell; DSC: decidual stromal cell; GM-CSF: granulocyte macrophage colony stimulating factor; IDO: Indoleamine 2,3-dioxygenase; MIF: Macrophage migration inhibitory factor; NK cell: natural killer cell; PBMC: peripheral blood mononuclear cell; PGE2: prostaglandin 2; TRAIL: TNF-related apoptosis-inducing ligand

Our interest focuses on decidual stromal cells (DSC) derived from the maternal side of the placenta, and amniotic epithelial cells (AEC), both of which have been shown to exert pleiotropic immune regulatory actions, mediated by complex mechanisms that inhibit the functions of different cell subsets of innate and adaptive immunity. A summary of their relevant features appears on Fig. 2.

DSCs play an important role in maintaining feto-maternal tolerance through an array of immunomodulatory mechanisms that are not completely elucidated. Although they have characteristics similar to those of bone marrow-derived MSCs, defined by the International Society for Cell Therapy (ISCT), DSCs are distinct from bone-marrow MSCs [31, 32]. Their chemokine release pattern (including TGFβ, IL-10, and MCP-1) inhibits NK cell proliferation, toxicity and IFN-γ production, and fosters their differentiation into a decidual NK (dNK) phenotype, with inhibitory and tissue repair properties. DSCs also secrete IL-33, contributing to the establishment of a Th2 microenvironment. Finally, they drive CD14 + monocyte differentiation to a tolerogenic dendritic cell phenotype, with low CD80 and CD86 expression, and IL-10, but not IL-12 production [33, 34].

In recent years, successful attempts at treating immune-mediated inflammatory diseases, such as graft versus-host disease (GVHD), by intravenous injection of decidual stromal cells (DSCs) have been reported in clinical trials from the Karolinska Institute. Control of the cytokine storm associated with this lethal condition and stimulation of tissue regeneration were achieved in these trials [35, 36]. The clinical safety of the systemic injection of DSCs was documented and its safety profile established [36, 37]. Of special interest, in the context of COVID-19 ARDS, in vivo cell tracking showed that DSCs migrated to the lungs and remained there for at least 48 h, where they could maintain protection and restoration of alveolar epithelial cells, reverse fibrosis and improve lung function [38]. In a case report from the same group, a patient suffering from sepsis-induced ARDS was treated with DSCs with a spectacular and fast response, leading to total weaning of oxygen supply within four days and a decrease of circulating inflammatory markers within a few hours [39].

Another key cell population for feto-maternal tolerance, AECs exhibit at least equally potent immunomodulatory features and appear to use wider-ranging mechanisms compared with other MSC types. They express and/or secrete FasL and non-classical MHC class 1 HLA-G, which bind to NK and T cells to trigger apoptosis and inhibit T-cell activation and proliferation [40–42]. They promote T-cell differentiation toward the Treg phenotype, which in turn induces a phenotype switch from M1 to M2 macrophages with tissue repair function [43, 44]. AECs also release anti-inflammatory cytokines and proteins, including IL-1 receptor antagonist, tissue inhibitors of matrix metalloproteinases − 1, -2, -3, and − 4, TGF-β and IL-10 [44, 45]. These soluble factors contribute to the regulation of macrophage recruitment and inhibit the chemotactic activity of neutrophils and macrophages [46]. In contrast to high expression of HLA-G, AECs express low levels of MHC class I antigens, while MHC class II antigens and costimulatory molecules are not expressed, protecting them from rejection [40].

Human AECs have shown efficiency in murine models of pulmonary fibrosis and preclinical models of broncho-pulmonary dysplasia [47, 48]. These effects were obtained through a lowering the number of pulmonary leucocytes and expression of pro-inflammatory markers (e.g., TGF-β, PDGF-α, PDGF-β, TNF-α, IFN-γ, and IL-6) and a Treg-induced phenotype switch in macrophages from M1 to M2 [43]. A recent, first-in-human, phase 1 trial in 7 premature infants from Australia has demonstrated the safety of systemic AEC administration, and the same group has published the protocol for a further study including efficiency endpoints [49–51].

It is well documented that MSCs exert most of their anti-inflammatory properties through their secreted extracellular vesicles (EV) and that EVs are an attractive and more versatile alternative to whole cell therapy, including for COVID-19 [52]. EVs derived from DSCs or AECs could be utilized to this end.

Viral transmission from mother to fetus seems to be extremely rare [53–55]. Reasons why the SARS-CoV-2 virus is not transmitted in utero may pertain to two issues: the placental barrier between mother and fetus, and the mechanisms by which the virus enters the infected cells. The prerequisite for cell infection by the SARS-CoV-2 virus is the identification of the angiotensin-converting enzyme-2 (ACE2) receptor by its spike protein. Only cells expressing ACE2 can get infected. In addition to ACE2, the cellular protease TMRRSS2 is also required to allow entry of the virus into host cells [56]. In a recent investigation of SARS-CoV-2 entry factors in multiple scRNA-seq datasets from different tissues, ACE2 was found to be expressed in cells derived from multiple tissues including airways, but at low levels. In contrast TMPRSS2 was highly expressed and had broader distribution, suggesting that the limiting factor for viral infection is ACE2 [57]. Interestingly, in placental or decidual tissues, ACE2 expression was only noticeable in very few cell types, but no TMPRSS2 expression could be detected, suggesting that DSCs and AECs may be protected from SARS-CoV-2 infection [47]. Indeed, a case of SARS-CoV-2 placental infection was recently reported, showing predominant location of the virus in the syncytiotrophoblast [58].

## The Case for DSC- or AEC-based Cell Therapy in Severe COVID-19 Disease

The characteristics reviewed above make some of the placenta-derived cells attractive candidates for the treatment of COVID19 pneumonia. Several factors contribute to make these cells more attractive than other MSC-derived cell products, including: (i) human placenta and fetal membranes are a perennial source of cells; (ii) they are available with limited ethical issues, since they are obtained from tissues discarded after childbirth; (iii) unlike bone marrow or adipose MSCs, no invasive procedure is needed for their recovery; (iv) large quantities of cells can be obtained from a single placenta and banked for repeated or multiple therapeutic use; (v) they migrate to the lungs after systemic injection; (vi) their low immunogenicity profile protects them from rapid immune destruction and dampens local activation of immunity; and (vii) they may be protected from SARS-CoV-2 infection.

With the existing clinical evidence for the innocuousness of the systemic administration of DSCs or AECs, we advocate for the initiation of clinical trials using this strategy. In order to avoid the controversy that has surrounded a number of previous clinical studies addressing the COVID-19 disease, we believe that such trials should take the form of RCTs, with robust efficacy endpoints. The injected cell dose should be based on previous clinical experience in GVHD and ARDS. Timing of administration of the cell therapy product should be carefully determined, early enough to tackle the cytokine storm before it becomes uncontrollable, but paying attention not to interfere with the host’s immune system while it is still able to mount a response against the SARS-CoV-2 virus. Clinical scores, such as the HScore, might be the right tools to help identify the moment to initiate therapy [10].

Finally, there is a need for proper determination of isolation techniques and means of characterization of the isolated cells. Further studies are also required to fully understand the potential of these cells in order to turn them into certified cell products, generated under good manufacturing practices (GMP), and authorized by the European Medicines Agency (EMA) in Europe and by the FDA in the United States.
